# Medial Position and Counterclockwise Rotation of the Parietal Scalp Hair-Whorl as a Possible Indicator for Non-Right-Handedness

**DOI:** 10.1100/tsw.2008.113

**Published:** 2008-08-31

**Authors:** Heinrich Schmidt, Martin Depner, Michael Kabesch

**Affiliations:** ^1^ Department of Medical Genetics, Dr. Von Hauner Children's Hospital, University of Munich, Lindwurmstr. 4, 80337 Munich, Germany; ^2^ Department of Pediatric Pneumology, Dr. Von Hauner Children's Hospital, University of Munich, Lindwurmstr. 4, 80337 Munich, Germany; ^3^ Community Paediatric Practice, Fichtenstr.27, 85220 Fuerstenfeldbruck, Germany

**Keywords:** parietal whorl, position, swirling direction, handedness

## Abstract

The objective of our study was to assess the association between position and swirling direction of the parietal whorl (PW) and handedness. In 519 patients of a pediatric practice, PWs were located and the swirling direction determined. Of those patients, handedness could be specified in 217. The right-sided PW (n = 347; 70.8%) and the clockwise (CW) swirling type (n = 411; 83.9%) of all participants were predominant in children with one PW. Non-right-handedness (NRH) was found in 40 (18.4%). Medial position of the whorl per se increases the chance for NRH, indifferent of the swirling direction. In patients with counterclockwise (CCW) swirling, the chance of NRH increased 3.5-fold for the right-sided, 5.4-fold for the left-sided, and 12.9-fold for the medial-positioned whorl. We conclude that NRH is associated with the position (medial!) and the swirling direction (CCW!) of the PW.

### INTRODUCTION

There is a strong relationship between early brain development and scalp hair patterning, both being of ectodermal origin. Between the 10th and 12th fetal week, the brain and the hemispheres, especially, show fast development. Thereby, the cranial vault rapidly enlarges to a domelike shape.

The focal point from which the skin starts to expand over this domelike outgrowth corresponds to the position of the posterior parietal whorl (PW). Because the hair follicles grow downward first, the stretch of the surface skin is greater than that of the underlying tissues, thus the hair follicles lag behind. This sloping angulation determines the hair directional patterning[1]. It is considered as a sign of lateralization.

In the past, in contrast to handedness, hair patterning has not been considered to be under genetic control. Handedness is considered to be the result of genetic and environmental factors[2,3,4,5]. Klar[3] was the first to observe the association between handedness and scalp hair-whorl rotation, suggesting a genetic model ("random-recessive model") with a single gene controlling both handedness and hair whorl orientation[3,6,7]. These findings were both confirmed and questioned by many other researchers[8,9,10]. However, no data exist regarding the position of the PW as an indicator for handedness.

The aim of the following study was to investigate a homogeneous German pediatric population with respect to the structural (PW position and swirling direction) and functional lateralization (handedness), focusing especially on the position of the PW.

### METHODS

From a community pediatric practice of the South German town of Fuerstenfeldbruck (40,000 inhabitants), patients seen for regular medical check-ups, common colds, or vaccinations were recruited for the study. Inclusion criteria were (1) both parents of Caucasian descent; (2) no signs of dysmorphism; (3) normal mental, motor, and auxologic development; (4) age: 6 months to 16 years. For the subgroup in which handedness could be determined, age was between 4 and 16 years. Exclusion criteria were (1) non-Caucasian descent (one or both parents), (2) dysmorphic features, (3) genetic disease, (4) mental or motor retardation, (5) microcephaly, (6) long hair with uncertain determination of the position of the PW/swirling pattern.

Data were collected by a single investigator. Dorsal head examination was performed with the patient sitting on a chair to ascertain position (left, medial, or right) and swirling direction (clockwise [CW] or counterclockwise [CCW]) of the PWs (see Fig. 1). Handedness was assessed by the Edinburgh handedness inventory, which ranges from –100 for strong left handedness to +100 for clear right handedness[11]. In this questionnaire, participants (or their parents) were asked which hand they used to write, to draw, to throw a ball, to use scissors, a toothbrush, a knife, a spoon, or a broom, to strike a match, or to open a box. Right handedness (RH) was considered if the Oldfield score was at least +30 and non-right-handedness (NRH) if the score was less than +30. Thus, left handers and ambidextrous children are pooled as non-right-handers.

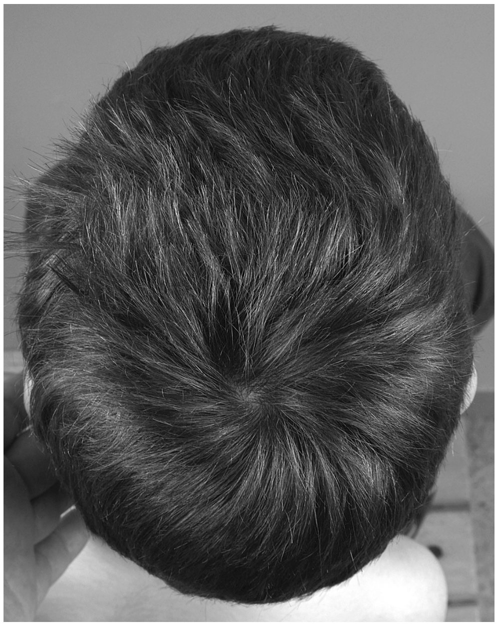

**FIGURE 1.** Dorsal head examination: medial position and CW rotation of the hair whorl.

In 519 children, the PW position as well as the swirling direction could be clearly ascertained. Handedness could be determined and classified in a subgroup of children with one whorl (n = 217). For statistical analysis regarding the association of handedness and PW, only patients with one whorl were considered.

All parents gave their informed consent prior to participation, according to the declaration of Helsinki.

### Statistical Analysis

To test the associations between handedness and characteristics of the PW, Chi-Square-tests were used. For the analysis that combines characteristics of PW, a logistic regression model, with the most common group as the reference group, was applied. Odds Ratios, with 95% confidence intervals and *p*-values were calculated. All tests were two-sided and a significance level of alpha = 0.05 was used. The analyses were performed with SAS 9.1.3.

## RESULTS

- **PW position** — A single PW was found in 490 (94.4%) and two PWs were found in 29 (5.6%) of the 519 participants (198 girls = 38.2%, 321 boys = 61.8%), respectively. In the patients with one whorl only, right-sided position was predominant (n = 347; 70.8%), followed by left-sided and medial position (n = 103; 21% and n = 40; 8.2%), respectively. If there were two whorls, one was usually on the right, the other on the left side of the head (data not shown) (Table 1).

**TABLE 1**
**Position of the Single PW in 490 Patients**

| | N (%) | Female Patients (%) | Male Patients (%) |
|---|---|---|---|
| Right | 347 (70.8) | 139 (40) | 208 (60) |
| Left | 103 (21) | 40 (38.8) | 63 (61.2) |
| Medial | 40 (8.2) | 11 (27.5) | 29 (72.5) |
| Total N | 490 (100) | 190 | 300 |

- **PW swirling direction** — In patients with a single whorl, swirling direction was CW in 411 (83.9%) and CCW in 79 (16.1%) participants, respectively. In children with two whorls, the right whorl swirls CCW and the left whorl CW. There were no significant differences between boys and girls regarding orientation or position of the PW (Table 2).

**TABLE 2**
**Swirling Direction of the Single PW in 490 Patients**

| | N (%) | Female Patients (%) | Male Patients (%) |
|---|---|---|---|
| CW | 411 (83.9) | 165 (40.1) | 246 (59.9) |
| CCW | 79 (16.1) | 26 (32.9) | 53 (67.1) |
| Total N | 490 (100) | 191 | 299 |

- **Handedness** — Handedness could be defined in a subgroup of participants (n = 217) (41.8% of all children with one PW) (not stratified for sex). NRH was found in 40 (18.4%) of them.
- **Handedness and swirling direction** — Regardless of the position of the whorl, NRH was found in 27 (14.5%) with CW and in 13 (41.9%) participants with CCW. Thus, there is about a threefold increase in NRH among children with CCW rotating whorls compared with the group of patients having a CW rotating whorl (Odds Ratio and 95% Confidence interval: 4.25 [1.87–9.67]; $p = 0.0003$).
- **Handedness and PW position** — Regardless of the swirling direction, NRH was found in 23 (16%) participants with right-sided, 9 (16.4%) with left-sided, and 7 (40%) with medial-positioned single whorl. Thus, there is a significant increase in NRH in the group of patients with medial-positioned PW (Table 3). Interestingly we could not find any difference between the right and left side, but a strong increase for NRH from right or left side to the medial position (Odds Ratio and 95% Confidence interval: 4.17 [1.53–11.39]; p = 0.003).

**TABLE 3**
**Frequency of NRH and RH in Regard to Position and Swirling Direction of the PW in 217 Patients**

| | Right Side | Median | Left Side | CW | CCW |
|---|---|---|---|---|---|
| NRH (N = 40) | N = 23 (16%) | N = 7 (40%) | N = 9 (16.4%) | N = 27 (14.5%) | N = 13 (41.9%) |
| RH (N = 177) | N = 121 (84%) | N = 10 (60%) | N = 46 (83.6%) | N = 159 (85.5%) | N = 18 (58.1%) |
| Total (N = 217) | N = 144 (100%) | N = 17 (100%) | N = 55 (100%) | N = 186 (100%) | N = 31 (100%) |

Taking together, CCW rotation and centrally located PW increases the chance for NRH markedly. In a final analysis, we used the combined groups (combined for orientation and location of the PW) and looked for the risk effects in so-called "risk groups". Results of logistic regression show that combinations significantly increase the risk for NRH (especially CCW and medially located PW) and that the results are more pronounced than for one risk factor (Table 4.).

**TABLE 4**
**Association of Handedness and Combinations of Properties of the PW (Position and Swirling Direction) in 217 Patients**

| | Whorl Position | Whorl Swirling Direction | Patients with Determinable Handedness | Effect for NRH (Odds Ratio and 95% Confidence Intervals) | *p* Value (Wald-Test) |
|---|---|---|---|---|---|
| Group 1 | Right | CW | 127 (58.5%) | 1.00 | |
| Group 2 | Right | CCW | 17 (7.8%) | 3.53 (1.15–0.80) | 0.027 |
| Group 3 | Left | CW | 44 (20.3%) | 0.65 (0.21–2.04) | 0.457 |
| Group 4 | Left | CCW | 11 (5.1%) | 5.39 (1.48–19.63) | 0.010 |
| Group 5 | Medial | CW | 15 (6.9%) | 4.31 (1.36–13.66) | 0.012 |
| Group 6 | Medial | CCW | 3 (1.4%) | 12.94 (1.11–150.59) | 0.040 |

### DISCUSSION

By 18 postconceptional weeks, hairs are extruded onto the surface of the head and face[1,12,13]. The three major foci from which the hair streams originate over the upper face and scalp are the two ocular point regions and the parietal hair whorl. From each ocular point, the frontal hair stream sweeps upwards in a lateral direction and from the posterior whorl, the hair streams flare out in all directions, thus the crossing of the two streams meet somewhere in the midforehead. It is hypothesized that hair directional patterning is mainly determined by brain growth, especially by hemisphere development.

There are few data regarding inheritance of the PW, but the high percentage of the right-sided and CW swirling type indicates genetic determination[3,14].

For "inheritance" of handedness, a purely acquired behavior theory, a single gene genetic model, or a gene-culture evolutional model have been proposed[2,3,15].

Klar[3] was the first to explore and find an association between handedness and the development of the PW: in a general population sample, consisting of mostly RH individuals, CCW whorl rotation was infrequent (8.4%), and NRH displayed a random mixture of CW and CCW swirling patterns. Re-examining this finding, in an independent sample of individuals chosen because of their CCW rotation, 50% were NRH. He concludes that a single gene controls handedness and whorl orientation, and proposes a "random-recessive model": two alleles on one locus control both handedness and hair-whorl orientation. The dominant allele predisposes to RH and CW hair orientation. Thus, a single copy would yield a right-handed preponderance. However, homozygosity for the recessive allele would result in a random distribution of RH and NRH. In addition, the "random allele" would lead to a separate random chance of having CCW hair-whorl rotation. Jansen et al.[9] challenged this genetic model, while finding CW rotation in about 80% of NRH and RH alike, as well as a lack of association between hemispheric language dominance, another sign of functional lateralization, and PW swirling direction.

There were far more boys than girls included in the study group for two reasons: (1) more boys than girls are prevalent in the outpatient setting and (2) girls usually have long hair, which often makes the items difficult to ascertain. However, this is only of marginal interest because a gender-specific difference regarding position or rotation of the PW is unlikely and could not be detected in our data.

Unfortunately, handedness could be defined in only 217 (41.8%) of all enrolled children (main exclusion criteria for the subgroup was age <4 years). The relatively small number of patients, especially of those with medial whorl in the subgroup, limits the statistical power and further research is required to confirm our results.

The most intriguing results in regard to our hypothesis were:

- NRH is associated with the medial position of the whorl, but not with the right- or left-sided position.
- NRH is associated with the CCW swirling direction.
- NRH is associated with the combination of position (especially medial) and CCW swirling direction.

Of special interest were the few patients with the medial whorl. At this point, we would like to remark that in three very young children (not included in the study population) with medial-positioned and CCW rotating whorl, we could successfully predict NRH.

Two questions remain to be answered: (1) why does the major part of the population have a right-sided CW swirling PW and a dominant left hemisphere with RH, and (2) what is the genetic basis of PW position and swirling direction? Due to the actual hypothesis, one can answer that the choice of dominant hemisphere, handedness, and swirling direction of the PW are genetically determined and the allele frequency will determine the preponderance of both traits[7].

Due to the presented data and the actual genetic and embryologic hypothesis, one may speculate that at the fetal stage, the right-handed individual exhibits rapid domelike bulging of the left-dominant cerebral hemisphere in the dorsolateral direction, thus inducing physical forces on the surface of the scalp followed by sloping of the hair shafts in the same direction, again followed by a CW swirling orientation when the nondominant, right hemisphere starts to grow in the same manner. Thus, an alternating outgrowth of dominant and nondominant hemisphere should occur under genetic control[3,14]. This hypothesis does not explain why some have two or more PWs.

We believe that thanks to these findings, a new jigsaw – the medial position of the PW as an indicator for NRH – could be added to the discovery of the association of structural and functional lateralization. These are the first data to reflect the importance of the position of the PW as an indicator for functional lateralization.

### CONCLUSION

The right-sided and CW rotating PW is predominant in humans; NRH is associated with CCW rotation regardless of the whorl position, but NRH is most prevalent in individuals with medially positioned and CCW rotating PW.

### ACKNOWLEDGMENTS

We thank Professor J.U. Walther for reviewing the manuscript.
